# Collagen and Elastic Fiber Content Correlation Analysis between Horizontal and Vertical Orientations of Skin Samples of Human Body

**DOI:** 10.1155/2015/692196

**Published:** 2015-09-14

**Authors:** Naveen Kumar, Pramod Kumar, Satheesha Nayak Badagabettu, Ranjini Kudva, Sudarshan Surendran, Murali Adiga

**Affiliations:** ^1^Department of Anatomy, Melaka Manipal Medical College, Manipal University, Manipal Campus, Manipal 576104, India; ^2^King Abdul Aziz Hospital, Al Jouf 42421, Saudi Arabia; ^3^Department of Pathology, Kasturba Medical College, Manipal, Manipal University, Manipal 576104, India; ^4^Department of Physiology, Kasturba Medical College, Manipal, Manipal University, Manipal 576104, India

## Abstract

*Background*. Unequal distribution of dermal collagen and elastic fibers in different orientations of skin is reported to be one of the multifocal causes of scar related complications. Present study is to understand the correlation pattern between collagen in horizontal (C_H_) and in vertical (C_V_) directions as well as that of elastic in horizontal (E_H_) and vertical (E_V_) directions. *Materials and Method*. A total of 320 skin samples were collected in two orientations from suprascapular, anterior chest, lateral chest, anterior abdominal wall, and inguinal regions of 32 human cadavers. Spearman correlation coefficient (*r*) was calculated between the variables (C_H_, C_V_, E_H_, and E_V_). *Results*. Significant positive correlation between C_H_ and C_V_, and between E_H_ and E_V_ observed in all 5 areas tested. A negative correlation between C_V_ and E_V_ at suprascapular, lateral chest, and inguinal regions and negative correlation between C_H_ and E_H_ at anterior chest and anterior abdominal wall have been identified. *Conclusion*. Knowledge of asymmetric content of dermal collagen and elastic fibers together with the varied strength and degree of association in the given area provides guidelines to the dermatologists and aesthetic surgeons in placing elective incisions in the direction maximally utilizing the anatomical facts for aesthetically pleasing result.

## 1. Introduction

The dermis of the skin has two parts that are accountable for sustaining the epidermis. The papillary part of the dermis is a thin sheet located just below the epidermis. It contains a small number of collagen and elastic fibers. The reticular layer of dermis lies deep into it, which contains larger bundles of collagen and elastic fibers that usually run parallel to surface of the skin.

Skin incisions or injury to the skin is known to produce scar. Hence, appearance of a scar, following wound repair, is a natural process. Intercomplimentary functions of collagen and elastic fibers play a key role in the wound healing and its subsequent consequences resulting in the formation of the scar. Sherratt reported that the types of collagen present in the scar tissue and the normal skin are similar, but with the diverged pattern of arrangement and distribution as compared to normal [1]. Similarly, Rnjak et al. reported the useful role of dermal elastic fibers in the process of wound healing and improvement of scar appearance [2].

Hypertrophic scars more commonly affect young individuals. Females are more prone to hypertrophic scars when compared to males for unknown etiology. The anterior chest, presternal region, shoulders, and deltoid regions are predisposed to hypertrophic scars [3–7]. Anterior chest area is also vulnerable for keloid formation [8]. Nevertheless, scar stretching occurs most frequently in the lower third of the scar overlying the xiphisternum and it may extend onto the abdomen [3].

In this study, we aimed to analyze the possible correlation between collagen and elastic fiber content in dermis of skin samples obtained from horizontal and vertical directions from selected areas of body and its possible effect in scar related complications.

## 2. Materials and Method

Three hundred and twenty (320) skin samples (160 each in horizontal and vertical orientations) at 5 different areas of human body were obtained from 32 formalin embalmed adult cadavers of either gender, aged between 50–70 years. Before obtaining the samples, the gross appearance of the skin quality of the cadaver was examined thoroughly to ensure its healthy appearance. The samples were obtained in two different orientations (horizontal and vertical) at randomly selected areas with the following descriptions:

1. Suprascapular area: skin samples were obtained along the superior border of trapezius muscle overlying the scapula.
2. Anterior chest area: skin overlying body of the sternum (at the level of 3rd costal cartilage) along its midline was taken. The vertical section was obtained along the vertical axis of the sternum, while horizontal section was across the former.
3. Lateral chest area: samples from skin covering 8th rib at anterior axillary line were collected in both the directions.
4. Anterior abdominal wall: samples of skin were taken at infraumbilical part (1 cm below the umbilicus) of anterior abdominal wall, overlying midline of linea alba.
5. Inguinal region: skin casing the inguinal ligament was collected. Skin along the direction of the ligament (horizontal) and perpendicular to it (vertical) was collected.

### 2.1. Tissue Processing

All the skin samples were processed for the histological preparation and employed with a special stain known as Verhoeff-van Gieson stain. While picrofuchsin solution of van Gieson selectively stains the collagen fibers, the elastic tissue is readily penetrated by the Verhoeff's stain by the multipurpose role of iodine in the solution [9].

### 2.2. Image Procurement and Image Analysis

From the special stained sections, photomicrographs were taken from entire thickness of dermis (involving both papillary and reticular parts) in 3 different fields of vision using Progress capture Pro 2.1 Jenoptic microscopic camera under 20x magnification. A total of 960 images were collected and subjected to image analysis by “Tissue-Quant” software version 1.0. Semiquantitative measure of dermal collagen and elastic fibers was performed in terms of percentage area occupied by these tissue structures [10, 11].

### 2.3. Statistical Analysis

Spearman correlation coefficient (*r*) was computed using SPSS package (version 15.0) to determine the linear association between the variables. Correlation coefficient (*r*) values were obtained in accordance with the following combined variables:

- C_H_ and C_V_ (collagen content between horizontal and vertical directions).
- C_H_ and E_H_ (between collagen and elastic fibers in horizontal direction).
- C_V_ and E_V_ (between collagen and elastic fibers in vertical directions).
- E_H_ and E_V_ (elastic fiber content between horizontal and vertical directions).

The results obtained by these calculations were interpreted according to the strength of association (strong, moderate, and low) and degree of correlation (positive or negative) according to reference table (Table 1) after taking into consideration the significant correlation (*p* < 0.01 or *p* < 0.05).

## 3. Results

**Figure 1:**
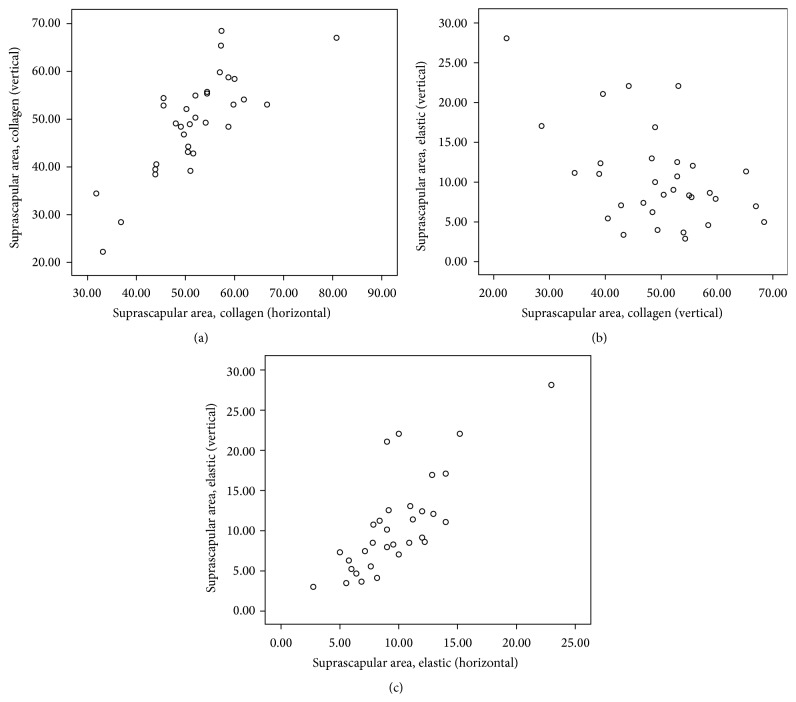
Scatter plot of significant Spearman correlation pattern at suprascapular area. (a) Strong positive correlation between C_H_ and C_V_, (b) low negative correlation between C_V_ and E_V_, and (c) strong positive correlation between E_H_ and E_V_ (C_H_: collagen in horizontal, C_V_: collagen in vertical, E_H_: elastic in horizontal, and E_V_: elastic in vertical direction).

**Figure 2 fig2:**
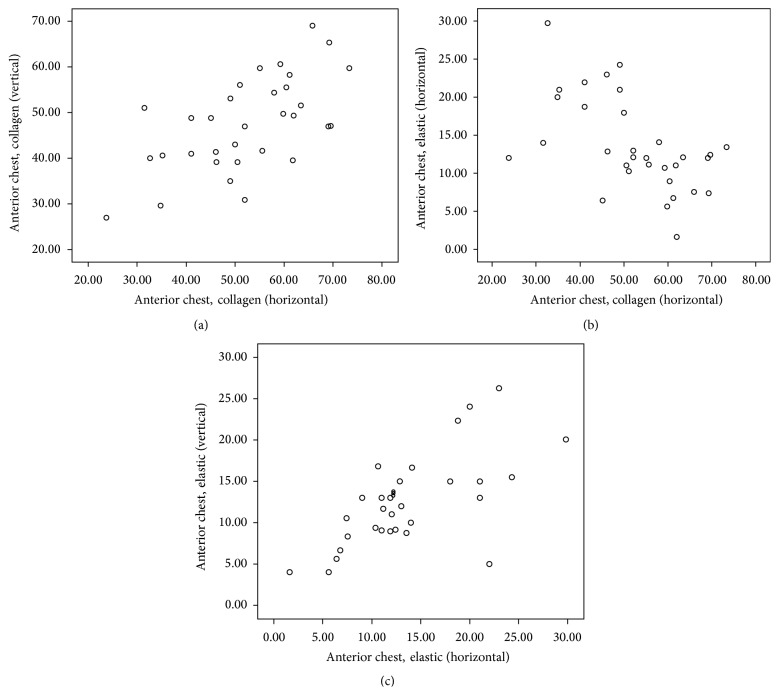
Scatter plot of significant Spearman correlation pattern at anterior chest area. (a) Moderate positive correlation between C_H_ and C_V_. (b) Moderate negative correlation between C_H_ and E_H_. (c) Moderate positive correlation between E_H_ and E_V_ (C_H_: collagen in horizontal, C_V_: collagen in vertical, E_H_: elastic in horizontal, and E_V_: elastic in vertical direction).

**Figure 3 fig3:**
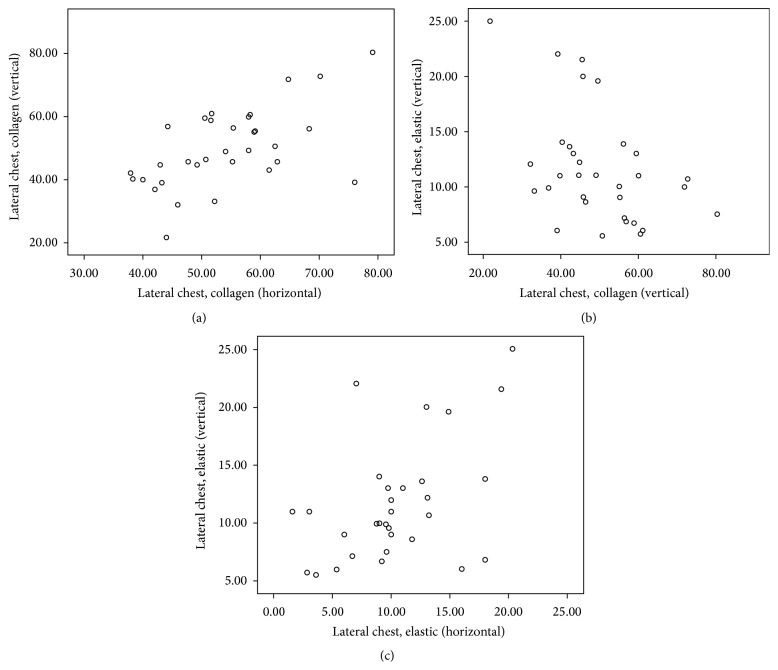
Scatter plot of significant Spearman correlation pattern at lateral chest area. (a) Moderate positive correlation between C_H_ and C_V_. (b) Low negative correlation between C_V_ and E_V_. (c) Low positive correlation between E_H_ and E_V_ (C_H_: collagen in horizontal, C_V_: collagen in vertical, E_H_: elastic in horizontal, and E_V_: elastic in vertical direction).

**Figure 4 fig4:**
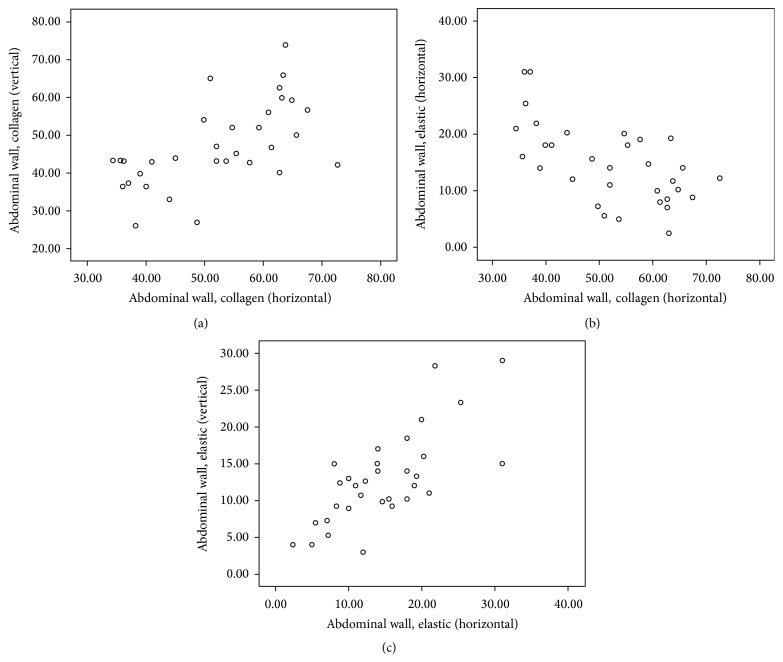
Scatter plot of significant Spearman correlation pattern at anterior abdominal wall. (a) Moderate positive correlation between C_H_ and C_V_. (b) Moderate negative correlation between C_H_ and E_H_. (c) Moderate positive correlation between E_H_ and E_V_ (C_H_: collagen in horizontal, C_V_: collagen in vertical, E_H_: elastic in horizontal, and E_V_: elastic in vertical direction).

Descriptive statistics of Spearman's rho correlation coefficient (*r*) and the level of significance (*p*) as computed from the selected areas of body are presented in Table 2 and corresponding scatter plots of significant (two-tailed) correlations are depicted in Figures 1–5. Comprehensive data with statistically significant (*p* < 0.01 or *p* < 0.05) correlation coefficient with the pattern of strength of association in each area are given in Table 3.

Spearman's correlation revealed a statistically significant positive correlation between C_H_ and C_V_ in all areas: suprascapular area (*r* = 0.75, *p* < 0.01), anterior chest area (*r* = 0.56, *p* < 0.01), lateral chest area (*r* = 0.55, *p* < 0.01), anterior abdominal wall (*r* = 0.57, *p* < 0.01), and inguinal region (*r* = 0.77, *p* < 0.01).

Similar to C_H_ and C_V_, statistically significant positive correlation was also noticed between E_H_ and E_V_ in all areas: suprascapular area (*r* = 0.77, *p* < 0.01), anterior chest area (*r* = 0.62, *p* < 0.01), lateral chest area (*r* = 0.43, *p* < 0.05), anterior abdominal wall (*r* = 0.69, *p* < 0.01), and inguinal region (*r* = 0.62, *p* < 0.01).

Significant negative correlations between C_H_ and E_H_ were seen only at anterior chest area (*r* = −0.55, *p* < 0.01) and in anterior abdominal wall (*r* = −0.57, *p* < 0.01).

Statistically significant negative correlation between C_V_ and E_V_ was also seen at suprascapular (*r* = −0.37, *p* < 0.05), lateral chest (*r* = −0.043, *p* < 0.05), and inguinal areas (*r* = −0.42, *p* < 0.05).

## 4. Discussion

The management of scar in the clinical setup is one of the most challenging tasks for aesthetic surgeons. This has not been resolved even after the incisions had been made on the skin in accordance with the standard lines of choice. The varied quantity and asymmetric content of dermal collagen and elastic fibers in different orientations of skin plane affecting the formation of scar remain a possibility [12].

Asymmetric distribution of dermal collagen and elastic fibers in different regions of human body is said to have regional discrepancies. It may be depending on the texture of the skin, as in head and neck region [13], anatomic, functional, or physical cause of skin tension as applicable in trunk region [10], and may be due to effect of various forces like burst force and stretch force exerted at joints [14]. These factors are found to be applicable in the management of wound healing consequences and scar management.

Selective incision on the skin was preferred to be line of Langer's. However, in some topographic sites of human body these lines are obscure and poorly differentiated. Conversely, role of an unequal distribution of dermal collagen and elastic fibers, as observable in two different orientations of the skin, is worth noticeable. Understanding the possible correlation between the collagen and elastic fibers between two different skin planes serves a valuable basis in the management of aesthetic procedures.

The correlation pattern analysis revealed that skin over the suprascapular area had strong positive correlation between C_H_ and C_V_ and between E_H_ and E_V_. Lateral chest showed moderate positive correlation between C_H_ and C_V_ and low positive correlation between E_H_ and E_V_. Inguinal region also showed strong positive correlation between C_H_ and C_V_ and moderate positive correlation between E_H_ and E_V_ contents. But all three areas exhibited low negative correlation between C_V_ and E_V_.

This indicates that the content of collagen and elastic fibers in these areas changes in accordance with their positive manner of correlation across two directions (horizontal and vertical), while, in the vertical direction, the content change between collagen and elastic fibers would be in reversed manner due to negative correlation between them.

In the remaining two areas, that is, anterior chest area and anterior abdominal wall, moderate positive correlation was observed between C_H_ and C_V_, and between E_H_ and E_V_. And in both areas, moderate negative correlation between C_H_ and E_H_ was observed. Therefore, in these areas, both the collagen and elastic fibers content between horizontal and vertical directions change in positive manner, but along the horizontal direction when collagen is increased corresponding elastic fiber content will be decreased and vice versa occurring consequently due to negative correlation effect.

Previous research study analyzing the unequal distribution of dermal collagen and elastic fibers across two directions in these areas reported that the abdomen area with higher content of both collagen and elastic fibers in horizontal direction accomplishes the functional cause factor together with Langer's line concept. Whereas the suprascapular, presternal, and lateral chest areas fulfill the dominance of functional cause factor over anatomical cause. Thus, in these areas, the collagen content in horizontal direction is significantly higher than vertical direction without any similar changes in their elastic fiber content between two directions. The groin area, on the other hand, witnesses the nullified effect of elastic fibers that necessitates its increased content in vertical direction without the changes of collagen content [10].

To summarize, significant positive correlation between C_H_ and C_V_ and between E_H_ and E_V_ as observed in all areas tested is dependent on the functional reason due to local stretch force effect rather than the anatomical cause factor as reported earlier [10]. This correlation pattern in these areas is in accordance with the collagen content pattern in two different directions as the collagen content is more in horizontal direction than in their vertical direction (C_H_ > C_V_).

On the other hand, negative correlation between C_H_ and E_H_ at anterior chest and anterior abdominal wall and negative correlation between C_V_ and E_V_ at suprascapular, lateral chest, and inguinal regions have been observed. This may be due to the fact that, in certain areas, constant active or passive stretch force works for the movements and gravity (as weight of upper limb which constantly produces rotation force on scapula/pulls the shoulder downward direction). The effect of these forces may be more in one or more directions which necessitates the requirement of increased elastic fiber content in that specific direction. Accordingly, the higher allotment of elastic fibers in horizontal direction than in the vertical direction (E_H_ > E_V_) was attributed to the areas where the negative correlation between C_H_ and E_H_ was noted, while reversal of this was seen in the remaining areas where the negative correlation between C_V_ and E_V_ exists.

## 5. Conclusion

Understanding the pattern of asymmetric content of dermal collagen and elastic fibers in a given area together with the stress on proposed incision in that area and degree of association between them in two different planes provides a valuable guideline to the dermatologists and surgeons in elective incisions that necessitates providing aesthetically pleasing results and in the management of scar related complications.

## Figures and Tables

**Figure 5:**
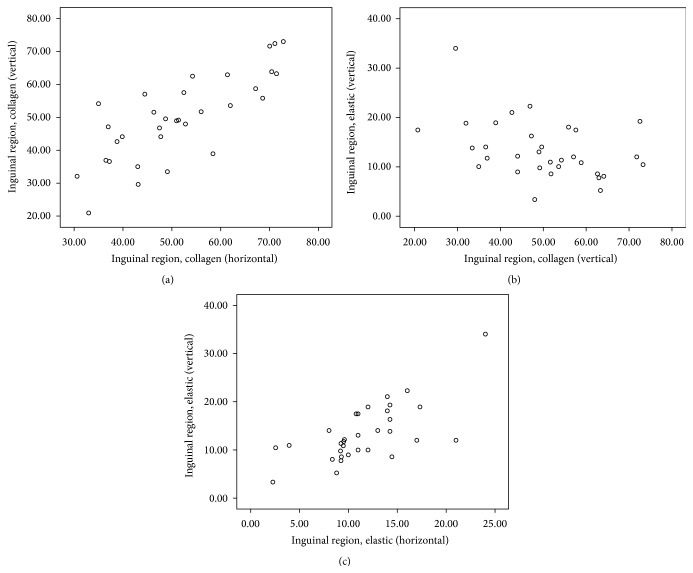
Scatter plot of significant Spearman correlation pattern at inguinal area. (a) Strong positive correlation between C_H_ and C_V_. (b) Low negative correlation between C_V_ and E_V_. (c) Moderate positive correlation between E_H_ and E_V_ (C_H_: collagen in horizontal, C_V_: collagen in vertical, E_H_: elastic in horizontal, and E_V_: elastic in vertical direction).

**Table 1:** Comparative description of correlation coefficient (*r*) and strength of association.

Correlation coefficient (*r*)	Strength of association
0.7 to 1 (positive)	Strong
−0.7 to −1 (negative)	

0.5 to 0.7 (positive)	Moderate
−0.5 to −0.7 (negative)	

0.3 to 0.5 (positive)	Low
−0.3 to −0.5 (negative)	

**Table 2:** Spearman's correlation coefficient (*r*) and its level of significance (*p*).

Variables between	Suprascapular area		Anterior chest area		Lateral chest area		Anterior abdominal wall		Inguinal region	
	*r*	*p*	*r*	*p*	*r*	*p*	*r*	*p*	*r*	*p*
C_H_ & C_V_	0.75	**0.000** ^#^	0.56	**0.001** ^#^	0.55	**0.001** ^#^	0.57	**0.001** ^#^	0.77	**0.000** ^#^
C_H_ & E_H_	−0.08	**0.62**	−0.55	**0.001** ^#^	−0.14	**0.41**	−0.57	**0.001** ^#^	−0.25	**0.15**
C_V_ & E_V_	−0.37	**0.03** ^**∗**^	−0.22	**0.21**	−0.43	**0.01** ^**∗**^	−0.33	**0.059**	−0.42	**0.01** ^**∗**^
E_H_ & E_V_	0.77	**0.000** ^#^	0.62	**0.000** ^#^	0.43	**0.01** ^**∗**^	0.69	**0.000** ^#^	0.62	**0.000** ^#^

^#^Correlation is significant at *p* < 0.01; ^*∗*^correlation is significant at *p* < 0.05.

(C_H_: collagen in horizontal, C_V_: collagen in vertical, E_H_: elastic in horizontal, and E_V_: elastic in vertical direction).

**Table 3:** Significant Spearman coefficient (*r*) and pattern of correlation trend profile.

	Between C_H_ and C_V_	Between E_H_ and E_V_	Between C_V_ and E_V_	Between C_H_ and E_H_
Suprascapular area	0.75 Strong +ve	0.77 Strong +ve	−0.37 Low –ve	—
Anterior chest	0.56 Moderate +ve	0.62 Moderate +ve	—	−0.55 Moderate –ve
Lateral chest	0.55 Moderate +ve	0.43 Low +ve	−0.43 Low –ve	—
Anterior abdominal wall	0.57 Moderate +ve	0.69 Moderate +ve	—	−0.57 Moderate –ve
Inguinal region	0.77 Strong +ve	0.62 Moderate +ve	−0.42 Low –ve	—

*r *= Spearman's correlation coefficient; +ve: positive correlation; −ve: negative correlation.

(C_H_: collagen in horizontal, C_V_: collagen in vertical, E_H_: elastic in horizontal, and E_V_: elastic in vertical direction).

## References

[1] Sherratt J. A. (2010). *Mathematical Modelling of Scar Tissue Formation*.

[2] Rnjak J., Wise S. G., Mithieux S. M., Weiss A. S. (2011). Severe burn injuries and the role of elastin in the design of dermal substitutes. *Tissue Engineering Part B: Reviews*.

[3] Elliot D., Cory-Pearce R., Rees G. M. (1985). The behaviour of pre-sternal scars in a fair-skinned population. *Annals of the Royal College of Surgeons of England*.

[4] Muir I. F. K. (1990). On the nature of keloid and hypertrophic scars. *British Journal of Plastic Surgery*.

[5] From L., Assad D., Jeffers J. D., Englis M. R. (1993). Neoplasms, pseudo-neoplasms and hyperplasia of supporting tissue origin. *Dermatology in General Medicine*.

[6] Hawkins H. K., Herndon D. N. (2007). Pathophysiology of the burn scar. *Total Burn Care*.

[7] Norman S. W., Christopher J. K. B., O'Connel P. R. (2008). Abnormal scars inflammation and abnormal wounds after healing. *Bailey and Love's Short Practice of Surgery*.

[8] Niessen F. B., Spauwen P. H. M., Schalkwijk J., Kon M. (1999). On the nature of hypertrophic scars and keloids: a review. *Plastic and Reconstructive Surgery*.

[9] Bancroft J. D., Gamble M. (2002). *Theory and Practice of Histological Techniques*.

[10] Naveen K., Pramod K., Satheesha N. B., Keerthana P., Ranjini K., Raghuveer C. V. (2014). Quantitative fraction evaluation of dermal collagen and elastic fibres in the skin samples obtained in two orientations from the trunk region. *Dermatology Research and Practice*.

[11] Prasad K., Kumar P. B., Chakravarthy M., Prabhu G. (2012). Applications of ‘TissueQuant’—a color intensity quantification tool for medical research. *Computer Methods and Programs in Biomedicine*.

[12] Naveen K., Pramod K., Keerthana P., Satheesha N. B. (2012). A histological study on the distribution of dermal collagen and elastic fibers in different regions of the body. *International Journal of Medicine and Medical Sciences*.

[13] Naveen K., Pramod K., Satheesha N. B., Keerthana P., Ranjini K., Raghuveer C. V. (2014). Histomorphometric analysis of dermal collagen and elastic fibers in skin tissues taken perpendicular to each other from Head and Neck region. *Journal of Surgical Academia*.

[14] Naveen K., Pramod N. B., Satheesha P., Keerthana K., Vasudevarao R. C. (2014). Surgical implications of asymmetric distribution of dermal collagen and elastic fibres in two orientations of skin samples from extremities. *Plastic Surgery International*.

